# Prediction of meat quality traits in the abattoir using portable near-infrared spectrometers: heritability of predicted traits and genetic correlations with laboratory-measured traits

**DOI:** 10.1186/s40104-021-00555-5

**Published:** 2021-03-12

**Authors:** Simone Savoia, Andrea Albera, Alberto Brugiapaglia, Liliana Di Stasio, Alessio Cecchinato, Giovanni Bittante

**Affiliations:** 1 Associazione Nazionale Allevatori Bovini di Razza Piemontese, strada provinciale Trinita’ 32/A, 12061 Carrù, CN Italy; 2 Department of Agronomy, Food, Natural Resources, Animals and Environment (DAFNAE), University of Padova (Padua), viale dell’Università 16, 35020 Legnaro, PD Italy; 3 Department of Agricultural, Forest and Food Sciences, University of Torino, Via L. Da Vinci 44, 10095 Grugliasco, TO Italy

**Keywords:** Genetic parameters, Meat quality, Near-infrared spectroscopy, Piemontese

## Abstract

**Background:**

The possibility of assessing meat quality traits over the meat chain is strongly limited, especially in the context of selective breeding which requires a large number of phenotypes. The main objective of this study was to investigate the suitability of portable infrared spectrometers for phenotyping beef cattle aiming to genetically improving the quality of their meat. Meat quality traits (pH, color, water holding capacity, tenderness) were appraised on rib eye muscle samples of 1,327 Piemontese young bulls using traditional (i.e., reference/gold standard) laboratory analyses; the same traits were also predicted from spectra acquired at the abattoir on the intact muscle surface of the same animals 1 d after slaughtering. Genetic parameters were estimated for both laboratory measures of meat quality traits and their spectra-based predictions.

**Results:**

The prediction performances of the calibration equations, assessed through external validation, were satisfactory for color traits (R^2^ from 0.52 to 0.80), low for pH and purge losses (R^2^ around 0.30), and very poor for cooking losses and tenderness (R^2^ below 0.20). Except for lightness and purge losses, the heritability estimates of most of the predicted traits were lower than those of the measured traits while the genetic correlations between measured and predicted traits were high (average value 0.81).

**Conclusions:**

Results showed that NIRS predictions of color traits, pH, and purge losses could be used as indicator traits for the indirect genetic selection of the reference quality phenotypes. Results for cooking losses were less effective, while the NIR predictions of tenderness were affected by a relatively high uncertainty of estimate. Overall, genetic selection of some meat quality traits, whose direct phenotyping is difficult, can benefit of the application of infrared spectrometers technology.

## Background

Improving meat quality attributes through genetic selection proved to be theoretically feasible as many quality traits display moderate to medium heritability values [1]. However, establishing a selection procedure depends on the availability of phenotypes collected as part of a routine recording scheme. This is a serious limitation when it comes to meat quality traits as it currently requires meat samples to be collected at the slaughterhouse, which depreciates the carcasses, while the subsequent laboratory analyses are expensive, and time consuming.

Visible and near-infrared spectroscopy (Vis-NIRS), which is based on the principle that different chemical bonds in organic matter absorb or emit light of different wavelengths when the sample is irradiated, offers a number of important advantages over conventional methods: ability to take rapid and frequent measurements, fast and simple or no sample preparation, suitability for on-line use, ability to simultaneously determine different attributes [2].

Several studies have assessed the use of reflectance spectroscopy to accurately predict the chemical composition of beef [3–5] and, with lower accuracy, different meat quality attributes [6–8].

However, with regards to genetic improvement, scientific knowledge is very scarce in beef cattle although genetic parameters of NIRS predictions of meat quality traits have been estimated in pigs [9]. In a previous study by our group [10], the only one that we are aware of to have made a genetic comparison between laboratory-measured and laboratory infrared-predicted meat quality traits of beef samples, we found medium-high genetic relationships between some of the measured and corresponding predicted meat quality traits. The genetic correlations for all the color traits and purge losses were high, and were greater than the corresponding phenotypic correlations, whereas both the phenotypic and genetic correlations for tenderness and cooking losses were negligible. These findings suggest the feasibility of genetically improving some meat quality traits using their NIR spectrometry predictions from meat samples. However, a selection program for meat quality traits could be more easily established if it were possible to routinely record phenotypes at the slaughterhouse without having to collect samples. In our previous study [10], meat samples were taken in the abattoir and after aging were transported to the laboratory where the muscle portions were dissected and minced; spectra were then acquired using a bench-top NIR spectrometer and laboratory analyses were carried out on the same sample, on the same day, and in the same laboratory.

The availability of new, portable NIR and Vis-NIR spectrometers able to collect spectra directly from the muscle surface at the slaughterhouse [7] means that we now have more efficient phenotyping tools for use in selection programs to improve meat quality traits.

In the absence of large-scale studies on the prediction of meat quality using portable NIR spectrometers, a research project (Qualipiem project) was set up with the aims of analyzing meat quality traits in Piedmontese young bulls, and of proposing innovative selection strategies for their improvement. The first steps taken were: to evaluate beef farming systems and other phenotypic sources of variation in carcass and meat quality traits using gold standard laboratory analyses [11]; to estimate their quantitative genetic parameters [12]; and to study their genome-wide associations and carry out pathway analyses [13]. In addition, two spectrometers very different in size, ease of use, and cost were compared for their ability to predict meat quality on the muscle surface in the abattoir without the need for meat sampling [14]. The obtained prediction accuracies showed a large variation, ranging from high values for most of the color traits to low values for meat tenderness and cooking losses. However, the prediction performance of calibration models was penalized because meat samples were affected by many processing factors prior to laboratory analyses (i.e. carcass aging and dissection, samples up-taking, transport and processing), which could not be predicted by spectra taken at the slaughterhouse. The conclusion of that study was that NIRS predictions of meat quality traits could be useful to capture the animal’s “native” characteristics, which is the case for the genetic improvement [14].

The main objective of this study was, therefore, to investigate the suitability of portable infrared spectrometers for phenotyping beef cattle as a basis for genetically improving the quality of their meat. The specific aims were: 1) to analyze the genetic variation in meat quality traits as predicted by two very different portable spectrometers on the intact cross-sectional muscle surface in the abattoir; 2) to compare this with the genetic variation in traits measured in the laboratory after sampling, aging, transport and analysis; 3) to assess the genetic relationships between laboratory measures of meat quality traits and their spectrum-based predictions.

## Methods

### Animals

The study was carried out on samples from 1,327 Piemontese young bulls slaughtered at the same commercial abattoir over 106 slaughter days. The young bulls were progeny of 204 A.I. purebred sires and 1,286 dams, all registered in the Italian Piemontese Herd Book.

The animals were fattened on 115 farms representative of the beef production systems in the Piemonte region (north-west Italy). The beef farming systems, feeding regimes, fattening conditions and slaughter performances of the young bulls are described in detail by Savoia et al. [11]. In brief, the young bulls were reared on farms operating one of the following beef production systems: traditional with restricted feeding and animals either kept in tie stalls or loose housed; modern breeders and fatteners or specialized fatteners using ad libitum feeding and loose housing (the last two systems were further subdivided according to whether or not they used total mixed rations).

The sampled young bulls had an average carcass weight of 438.1±43.6 kg, and an average age at slaughter of 541±63 days, giving an average daily carcass gain of 0.818±0.107 kg/d. Average carcass conformation score (using the SEUROP classification system with each category divided into 3 subclasses giving a scale of 18 points) was 14.66, corresponding to an average evaluation close to “E+” in the EU linear grading system; the average rib eye area measured at the 5th rib was 92±14.3 cm^2^.

### Spectra collection

Spectra collection and the technical characteristics of the instruments are described in detail by Savoia et al. [14]. Briefly, the spectra were acquired with the following spectrometers:

- Vis-NIRS: LabSpec 2500 (ASD Inc., Boulder, CO, USA), which has a spectral range in the visible and near-infrared sections of electromagnetic radiation (wavelengths 350 to 1830 nm), measurements taken every 1 nm producing 1481 data points per sample; the instrument’s dimensions are 12.7 cm × 36.8 cm × 29.2 cm, and it weighs 5,600 g; the spectra are collected with a probe (26 cm × 10 cm × 5 cm) connected to the instrument with an optical fiber;
- Micro-NIRS: Micro NIR Pro (JDSU San Jose, CA, USA), which has a spectral range in the near-infrared region (wavelengths 905 to 1,649 nm), measurements taken every 6 nm producing 125 data points per sample; the instrument’s dimensions are 4.5 cm × 4.4 cm × 4.0 cm, and it weighs 60 g; the spectra are collected directly by the instrument, which should be connected to a lap-top or tablet via a USB cable.

The right side of each carcass was divided into 2 quarters between the 5th and 6th ribs (pistol cut) in the abattoir the day after slaughter (about 24 h post-mortem). The spectra were collected on the cross-sectional surface of the longissimus thoracis muscle using the scanning head of the fiber-optic contact probe (10 mm in diameter) of the Vis-NIRS or by applying the Micro-NIRS to the surface of the muscle. Five spectra were acquired with each instrument from different positions on the cut surface of the same muscle.

### Beef sample collection and meat quality analyses

Twenty-four hours after slaughter and immediately following spectra collection, individual samples (4.0 cm thick) of the longissimus thoracis muscle were collected from between the 5th and 6th ribs, then were individually vacuum packed and transferred under refrigerated conditions to the laboratory, where they were stored in a chilling room at 4 °C for 6 d (meat aging), after which meat quality traits were measured on all samples.

Meat quality was assessed 7 d after slaughter by measuring: muscle pH in triplicate using a digital pH-meter; lightness (L*), redness (a*), yellowness (b*), hue angle (h*), chroma (C*) in triplicate after 1 h blooming using a Minolta CR-331C colorimeter; purge losses (PL, %) by difference between the weight of the sample cut at carcass dissection (day 1 after slaughtering) and before analyses (day 7); cooking losses (CL, %) by difference between of the meat subsample before and after cooking meat sample in a water-bath till an internal temperature of 70 °C; tenderness (Warner Bratzler shear force; WBSF, N) on 6 cylindrical 1.27 cm-diameter cores of cooked meat. Details of the procedures used to assess the meat quality traits can be found in Savoia et al. [11].

### Statistical analyses

#### Spectral data editing and validation procedure

The spectral data were edited and processed according to the model described in detail in the previous study by Savoia et al. [14]. In brief, records with errors (e.g., individually identified spectra not matching the reference samples) and outlier spectra identified by Mahalanobis distance were discarded from the two original spectral datasets obtained with the Vis-NIR and Micro-NIR portable spectrometers. Before developing the calibration equations, the spectral data were centered and standardized to improve the goodness of fit of the chemometric modeling.

A Bayesian model (Bayes B) implemented with the BGLR library [15] of the R-software was used to develop calibration equations for each beef quality trait, as described in detail by Ferragina et al. [16].

In order to reproduce operational conditions, an external validation procedure was carried out to predict meat quality traits from the calibration equations based on the Vis-NIR and Micro-NIR spectra. As the most important source of variation was the slaughter batch (all animals slaughtered on the same day), external validation consisted in predicting the observations of a given batch from the calibration equations developed using the measured meat quality data of animals slaughtered on all the other days (“leave-one-batch-out” procedure). This procedure has been repeated for each batch accumulating the predicted values. Therefore, the predictions of meat quality data for each animal were obtained using prediction equations developed without the observations measured on that animal and on all the animals slaughtered in the same batch. As a consequence, the entire set of predicted data could be used for the estimation of genetic parameters without any risk of inflation of the estimates. The R^2^ values of external validation have been used to evaluate the prediction accuracy of calibration models. The “leave-one-batch-out” procedure’s details are described in Savoia et al. [14].

The final dataset used to estimate (co)variance components and to evaluate the magnitude of the genetic correlations contained the measured and predicted observations on a number of animals ranging from 1,117 to 1,134 depending on the trait.

#### Estimates of (co)variance components and genetic parameters

(Co)variance components were estimated by REML procedures using the VCE software [17]. For each of the meat quality traits, (co)variance components were estimated through separate bivariate analyses including the measured trait and its prediction obtained with either the Vis-NIR or Micro-NIR spectrometer.

The general model can be written in matrix notation as:

$$ \mathrm{y}=\mathrm{X}\beta +\mathrm{W}\mathbf{1}\mathrm{c}+\mathrm{W}\mathbf{2}\mathrm{q}+\mathrm{Zu}+\mathrm{e} $$

where **y** contains the observations for traits 1 and 2, **β** is the vector of non-genetic fixed effects, **c** is the vector of random herd effects (98 levels), **q** is the vector of the random batch effect (106 levels), **u** is the vector of animal additive genetic effects, **e** is the vector of random residual effects, and **X**, **W1**, **W2** and **Z** are incidence matrices of proper dimensions. Fixed effects of parity of the dam (4 classes: 1st, 2nd, 3rd–8th, >8th) and age at slaughter (5 classes: < 450 d, 450–510 d, 511–570 d, 571–630 d, > 630 d) have been included in the model for pH and L*, respectively. Effects of different herds were assumed to be normally and independently distributed, **c** ~ N(0, **C** ⊗ **I**); effects of batch were assumed to be normally and independently distributed, **q** ~ N(0, **Q** ⊗ **I**). A minimum cell size of 3 observations was required for both herd and batch effects. Animal additive genetic effects were assumed to be normally distributed, **u** ~ N(0, **G** ⊗ **A**), where **G** is the (co)variance matrix between the animal effects, and **A** is the numerator of the Wright’s relationship matrix. Additive relationships were computed using a pedigree file that included the phenotyped animals and all their known ancestors (13,122 animals). Residuals were assumed to have a normal distribution, **e** ~ N(0, **R** ⊗ **I**).

To facilitate comparison with literature estimates, we estimated intra-herd heritability defined as:

$$ {\mathrm{h}}^2={\upsigma^2}_{\mathrm{a}}/\left(\ {\upsigma^2}_{\mathrm{a}}+{\upsigma^2}_{\mathrm{e}}\right) $$

where σ^2^_a_ is the additive genetic variance, and σ^2^_e_ is the residual variance. Intraherd heritability is equivalent to standard heritability under models treating contemporary groups (herds/batches) as fixed effects.

## Results

Descriptive statistics of the meat quality traits measured in the laboratory on aged samples according to gold standard methods, and their predictions obtained from the spectra acquired in the abattoir the day after slaughter are reported in Table 1.

**Table 1:** Descriptive statistics of meat quality traits^a^ and of their predictions, and calibration equations prediction performances

	L*	a*	b*	C*	h*	pH	PL, %	CL, %	WBSF, N
Carcasses, *n*	1,129	1,133	1,134	1,133	1,131	1,127	1,128	1,134	1,117
Laboratory measures									
Mean	39.86	28.59	9.65	30.19	18.53	5.55	4.50	16.76	40.96
SD	3.46	1.74	1.66	2.15	2.04	0.05	1.19	3.45	10.43
Vis-NIRS predictions									
Mean	39.87	28.60	9.63	30.18	18.50	5.56	4.47	16.80	40.89
SD	3.17	1.34	1.38	1.73	1.68	0.04	0.60	1.43	4.79
R^2^_EXT_^b^	0.84	0.55	0.63	0.58	0.64	0.30	0.31	0.16	0.16
Micro-NIRS predictions									
Mean	39.89	28.62	9.70	30.22	18.56	5.55	4.50	16.67	40.89
SD	3.12	1.22	1.29	1.56	1.61	0.02	0.53	0.75	3.84
R^2^_EXT_^b^	0.80	0.52	0.61	0.55	0.63	0.22	0.27	0.19	0.19

^a^ L* = lightness; a* = redness; b* = yellowness; C* = chroma; h = hue angle; *PL* purge losses (%), *CL* cooking losses (%), *WBSF* shear force (N). ^b^ R^2^_EXT_ = prediction performance of calibration equations evaluated through external validation

**Fig. 1:**
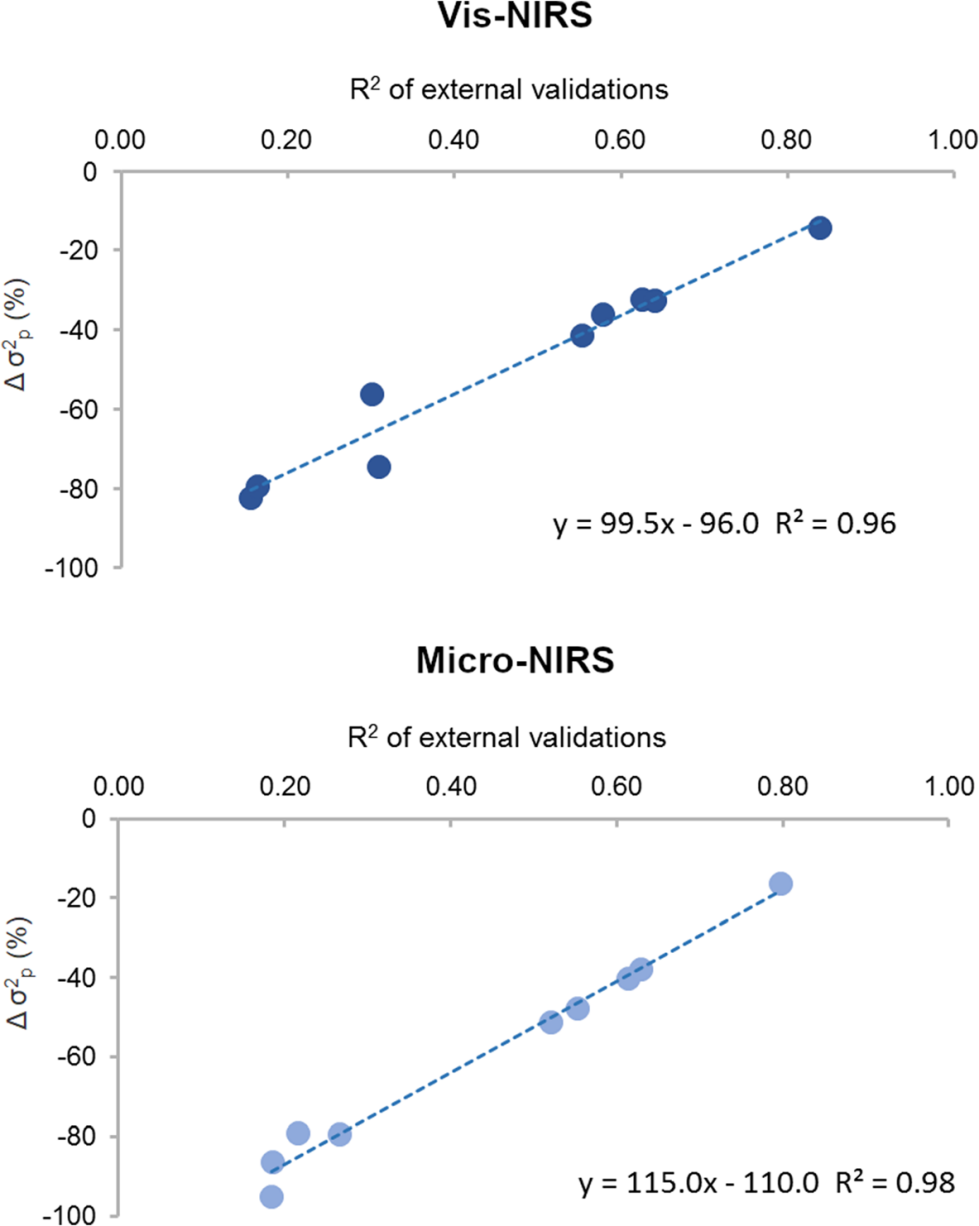
Relationship between R^2^ of external validations of predicted meat quality traits and the decrease in phenotypic variance (∆σ^2^_p_) of predictions compared with the laboratory measured traits

Large differences in variability across the traits measured in the laboratory were observed. Water loss traits (PL and CL) and WBSF showed the highest variability followed by color traits, whereas the SD of the pH measurements was very low. For all the traits considered, the average values of the predictions obtained with both instruments were very similar to the corresponding laboratory measurements. On the other hand, the variability in the predicted traits was always much lower than in the measured traits. This was particularly marked for PL, CL, and WBSF, where the standard deviation of the predictions was 50% to 78% lower than that of the measured traits. The reduction was less pronounced (− 10% to − 27%) for color traits. Loss of variability was in general more marked in the predictions obtained with the Micro-NIRS spectrometer (− 40%) than with the Vis-NIRS instrument (− 30%). The externally validated prediction performance of the calibration equations was satisfactory for all color traits (R^2^_EXT_ 0.52 to 0.80), low for pH and PL (R^2^_EXT_ around 0.30), and very poor for CL and WBSF (R^2^_EXT_ below 0.20). No relevant differences were observed between the two spectrometers in terms of the magnitude of R^2^_EXT_. Across traits, there was a clear relationship between loss of variability in the predictions with respect to the measurements and the quality of the prediction performance (R^2^ 0.96 for Vis-NIRS, R^2^ 0.98 for Micro-NIRS, Fig. 1).

Table 2 compares laboratory-measured and spectra-predicted traits with respect to the variance components and heritabilities of color traits. The batch effect was the most important source of variation for all traits, with the exception of L*, accounting for 15% to 30% of the total variance. The incidence of this effect was always lower in the Micro-NIRS predictions than in the Vis-NIRS predictions and the laboratory-measured traits. The effect of the fattening herd was small (5% to 10% of total variance according to the trait) and relatively homogeneous for the laboratory-measured and spectra-predicted traits.

**Table 2:** Variance components and intraherd heritability of color traits^a^ and of Vis-NIRS and Micro-NIRS predictions

	L*			a*			b*			C*			h*		
	Lab trait	Vis-NIRS	Micro-NIRS	Lab trait	Vis-NIRS	Micro-NIRS	Lab trait	Vis-NIRS	Micro-NIRS	Lab trait	Vis-NIRS	Micro-NIRS	Lab trait	Vis-NIRS	Micro-NIRS
Phenotypic variance	11.64	9.96	9.74	3.12	1.83	1.52	2.79	1.88	1.67	4.71	3.01	2.46	4.17	2.81	2.59
Variance components^b^															
Additive genetic	0.23	0.32	0.28	0.09	0.04	0.04	0.10	0.02	0.04	0.09	0.03	0.03	0.10	0.03	0.09
Batch	0.18	0.15	0.15	0.25	0.31	0.22	0.23	0.26	0.16	0.25	0.30	0.21	0.21	0.24	0.18
Herd	0.06	0.06	0.05	0.11	0.11	0.10	0.08	0.08	0.07	0.10	0.10	0.10	0.06	0.06	0.07
Residual	0.54	0.46	0.52	0.55	0.53	0.64	0.59	0.64	0.73	0.56	0.58	0.65	0.62	0.67	0.67
Intraherd heritability															
Estimate	0.30	0.41	0.35	0.14	0.08	0.07	0.14	0.04	0.05	0.14	0.04	0.05	0.14	0.05	0.11
SE	0.09	0.10	0.11	0.07	0.07	0.05	0.07	0.04	0.06	0.07	0.05	0.05	0.07	0.06	0.08

^a ^L* = lightness; a* = redness; b* = yellowness; C* = chroma; h = hue angle

^b ^Ratio to phenotypic variance

The animal additive genetic effect explained 20% to 30% of the total variance of L*, according to the instrument, with a higher incidence in the predicted than in the measured traits for both instruments. As a consequence, the intraherd heritability values were relatively high, ranging from 0.30 in the lab measurements to 0.41 in the Vis-NIRS predictions. All the other color traits behaved differently: the proportion of variance in the animal effect was around 10% and consistent across the measured traits, and was much lower with both prediction techniques. Heritability values for the predictions were therefore quite low compared with the heritability of 0.14 for the laboratory measured traits, and fairly homogeneous, with values ranging from 0.04 to 0.08 with the only exception of h* from the Micro-NIRS spectrometer.

The variance of the batch effect was very high for measured pH, CL and WBSF, and for most of the corresponding predictions, accounting for between 40 and 60% of total variance (Table 3). Only the Micro-NIRS predictions of CL exhibited a small variance in batch (13%). The variances in batch of both the measured and predicted PL were similar to those of color traits. Likewise, there was little variability in the meat quality traits due to the herd effect, in most of the cases not exceeding 7% of the total variance.

**Table 3:** Variance components and intraherd heritability of meat quality traits^a^ and of Vis-NIRS and Micro-NIRS predictions

	pH			PL, %			CL, %			WBSF, N		
	Lab trait	Vis-NIRS	Micro-NIRS	Lab trait	Vis-NIRS	Micro-NIRS	Lab trait	Vis-NIRS	Micro-NIRS	Lab trait	Vis-NIRS	Micro-NIRS
Phenotypic variance	0.30^c^	0.13^c^	0.06^c^	1.39	0.36	0.28	11.78	2.07	0.57	113.14	23.33	15.41
Variance components^b^												
Additive genetic	0.08	0.08	0.06	0.10	0.17	0.10	0.10	0.03	0.01	0.16	0.00	0.05
Batch	0.61	0.48	0.49	0.14	0.21	0.16	0.42	0.54	0.13	0.42	0.53	0.40
Herd	0.06	0.07	0.05	0.05	0.04	0.15	0.04	0.04	0.03	0.06	0.07	0.07
Residual	0.25	0.37	0.40	0.71	0.58	0.69	0.44	0.40	0.83	0.37	0.41	0.48
Intraherd heritability												
Estimate	0.25	0.18	0.13	0.13	0.22	0.13	0.19	0.07	0.01	0.31	0.00	0.10
SE	0.09	0.08	0.09	0.07	0.10	0.07	0.08	0.06	0.04	0.10	0.04	0.07

^a ^*PL* purge losses (%), *CL* cooking losses (%), *WBSF* shear force (N)

^b ^Ratio to phenotypic variance

^c^ × 10^2^

The proportion of variance explained by the additive genetic effect was much higher in the measured CL and WBSF than in their predictions. The heritabilities of the measured traits were moderate for CL (0.19) and relatively high for WBSF (0.31), but those of the predicted traits were considerably lower. In particular, the predictions of CL obtained with Micro-NIRS and WBSF obtained with the Vis-NIRS instrument showed an almost null incidence of additive genetic variance, and the resulting heritabilities were close to zero. For both pH and PL, the estimated heritabilities of the predictions obtained from the Vis-NIRS instrument were higher than those from the Micro-NIRS (0.18 vs. 0.13 for pH, 0.22 vs. 0.13 for PL). The heritability of measured pH was higher than that of the corresponding predictions, whereas the heritability of measured PL was similar to that of the Micro-NIRS predictions and markedly lower than that of the Vis-NIRS predictions.

Estimates of the genetic and residual correlations obtained by bivariate analyses of color and meat quality traits measured on aged meat samples in the laboratory, and their predictions obtained from meat spectra acquired in the abattoir by both the Vis-NIRS and Micro-NIRS are presented in Table 4. The values of the residual correlations reflect the prediction performance of the calibration equations. Genetic correlations between the lab-measured and spectra-predicted traits were always higher than the corresponding residual correlations. Their average value across traits was 0.81 compared with values of around 0.50 for the residual correlations for both instruments. Large differences in the genetic correlations among traits were observed. These were extremely high for color traits and PL, almost always 1.0 for the Vis-NIRS, and on average 0.9 for the Micro-NIRS. Among the other traits, the genetic correlations were of a lower magnitude and differed between the two spectrometers. The estimated genetic correlations obtained from the Vis-NIRS were greater than those from the Micro-NIRS, particularly for pH (0.70 vs. 0.45), CL (0.70 vs. 0.25) and WBSF (0.81 vs. 0.42).

**Table 4:** Additive genetic (r_a_) and residual correlations (r_e_) of color and meat quality traits with their predictions

Traits^a^	Vis-NIRS		Micro-NIRS	
	r_a_	r_e_	r_a_	r_e_
L*	1.000 (0.001)	0.871 (0.016)	1.000 (0.001)	0.831 (0.022)
a*	0.958 (0.173)	0.671 (0.029)	0.783 (0.225)	0.646 (0.031)
b*	1.000 (0.001)	0.761 (0.021)	0.930 (0.189)	0.598 (0.025)
C*	1.000 (0.001)	0.703 (0.024)	0.771 (0.228)	0.687 (0.027)
h*	1.000 (0.001)	0.763 (0.057)	0.858 (0.134)	0.756 (0.026)
pH	0.701 (0.164)	0.358 (0.056)	0.448 (0.256)	0.262 (0.028)
PL, %	0.979 (0.085)	0.385 (0.054)	0.879 (0.162)	0.378 (0.045)
CL, %	0.703 (0.168**)**	0.120 (0.059)	0.248 (0.271)	0.265 (0.058)
WBSF, N	0.805 (0.187)	0.202 (0.055)	0.418 (0.316)	0.271 (0.070)

^a ^L* = lightness; a* = redness; b* = yellowness; C* = chroma; h = hue angle; *PL* purge losses (%), *CL* cooking losses (%), *WBSF* shear force (N)

SE in parentheses

Overall, the prediction performances of the two spectrometers were quite similar for all the traits considered. The genetic parameters of the predictions for color traits were comparable, whereas the Vis-NIRS performed better than the Micro-NIRS in the other meat quality traits.

## Discussion

The main objective of this research was to evaluate the possible use of NIRS technology to predict phenotypes for meat quality traits for genetic evaluation purposes. The use of portable instruments at the slaughterhouse, and spectra acquisition from the muscle surface naturally exposed during routine subdivision of half-carcasses into quarters could eliminate the need for meat sample collection, which depreciates the carcasses, and for the subsequent transport, aging and laboratory analyses. In a recent work from the same project, we described, compared and discussed in detail the Vis-NIR and Micro-NIR spectroscopic techniques in terms of instrument characteristics, repeatability, calibration, cross-validation, and external validation of the predictions, and their ability to capture the main phenotypic sources of variation in meat quality traits [14]. In this study, discussion of the two spectroscopic techniques was focused on estimates of genetic parameters and their use for the genetic improvement of meat quality in beef cattle populations.

In our investigation the predictive performance of NIRS was evaluated using external validation, which led to R^2^ values lower than those in most of the published studies using random cross-validation. In other foods, Bittante et al. [18] and Eskildsen et al. [19] showed that the random cross-validation procedure tended to overestimate the predictive ability of FTIR predicted traits compared with external validation. This over-fitting is particularly large for traits affected by high environmental variation related to farms, seasons, or batches. Calibration model parameters, such as cross or external validation (R^2^ or RMSE), are not sufficient to establish the usefulness of spectral predictions for the purposes of genetic improvement. Although Soyeurt et al. [20] consider an R^2^ of cross-validation exceeding 0.75 to be necessary in order to use predictions of dairy traits for selection, some authors report satisfactory results with moderate or even low prediction performances for milk [18, 21] and meat quality traits [10]. Aside from R^2^, the suitability of infrared predictions as indicator traits for selection relies especially on a combination of their heritability and loss of additive genetic variance with respect to measured traits and their genetic correlations with the corresponding measurements [21, 22].

### Heritability of measured and predicted meat quality traits

The heritabilities of the laboratory-measured meat quality traits were in the range of most literature reports [23, 24], and were extensively discussed in our previous study using this dataset [12]. In a study on the genetic basis of meat quality traits in the Piemontese breed, Boukha et al. [1] found similar heritabilities to this study for L*, b* and C*, but their estimates were considerably higher for a* and h*. However, different tools were used to measure color traits in that study. The heritabilities of the other measured meat quality traits were in some cases lower (CL and WBSF), and in other cases higher (pH and PL) than in our study. The theoretical basis of the heritability of infrared predictions lies not only in the phenotypic correlations with meat quality traits (prediction accuracy), but also in the fact that the absorbance of electromagnetic radiation by the meat surface may be affected by the animal’s genetics, i.e., it may be heritable. Different sections of the milk infrared spectrum have been shown to have variable degrees of heritability [25, 26], but this has not so far been shown for meat.

In general, the predictions of meat quality traits obtained from spectra acquired in the abattoir yielded lower heritability values than the corresponding traits measured on aged meat samples in the laboratory, with the exception of L* and PL. The difference in heritabilities between the predicted and measured traits was on average 0.08 for both spectrometers, but there was variability across traits. Although the heritabilities of the meat quality predictions were lower than the heritabilities of the measured traits, in most cases they were large enough to be exploited for selection. Only the predictions of CL obtained with Micro-NIRS, and the predictions of WBSF obtained with Vis-NIRS were of no use, as their additive genetic variance was almost null. So far, our previous survey has been the only one to address the estimation of genetic parameters for meat quality traits predicted by NIR spectroscopy [10]. That investigation was carried out on cattle of the same breed and sex, and similar age to those in this study. However, the spectra were acquired from minced, aged samples with a bench-top spectrometer in laboratory conditions, and the calibration equations were developed using a different procedure based on partial least squares regression and random assignment of samples to the calibration and testing sets. Unlike the present study, the predictions of all the meat quality traits, except L*, had similar or greater heritabilities compared with the measured traits [10]. In general, the estimates of heritability of the predicted traits were higher than in the present study for most of the traits, and no null heritabilities were found also for CL and WBSF predictions. It should be borne in mind that in the previous study the predictions were obtained from spectra acquired on the same day and on the same material used for the meat analyses, and that the validation data were not entirely independent of the calibration data. In the present study, the spectra were acquired in the abattoir 6 d before the laboratory analyses, so these calibrations predict the quality of the meat after sampling, aging, transport, and analysis.

In this investigation, the lower heritability of the predictions compared with the measurements was due to the additive genetic variance being on average 70% lower, which exceeded the decrease observed in the phenotypic variance, ranging from 50% to 60%, depending on the instrument. These results are consistent with those of Bonfatti et al. [22], who found a similar pattern in the infrared predictions of a large number of traits related to milk composition and technological properties at the population level. On the other hand, other studies on the same traits reported that the lower additive genetic variance in the infrared predictions compared with the measured traits was also associated with a lower residual variance leading to increased heritability values, particularly for poorly predicted traits [18, 27].

**Fig. 2:**
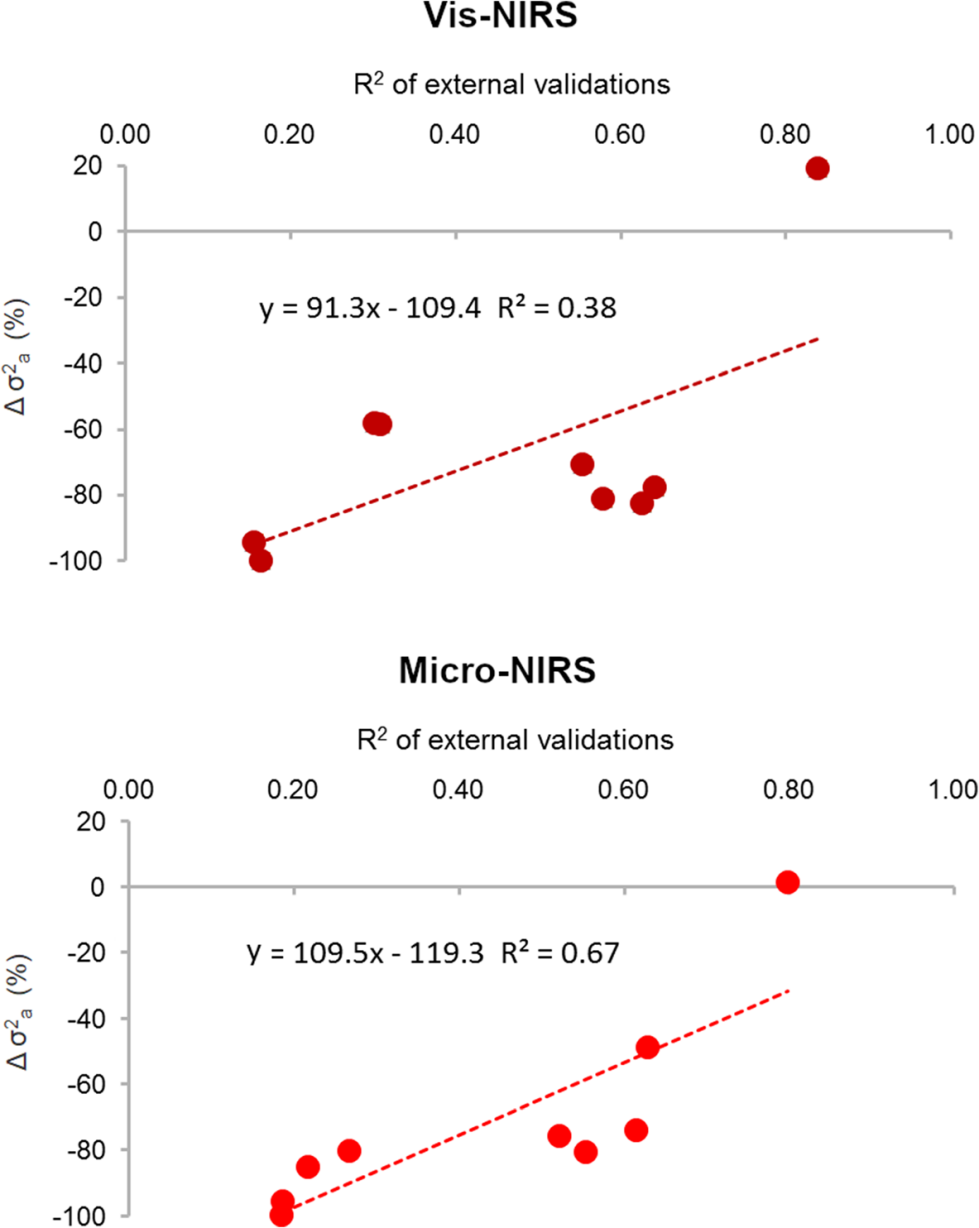
Relationship between R^2^ of external validations of predicted meat quality traits and the decrease in additive genetic variance (∆σ^2^_a_) of predictions compared with the laboratory measured traits

The lower phenotypic variability in the meat quality predictions observed in this study was strongly related to the accuracy of the calibrations measured by the R^2^ of the external validations (Fig. 1). As expected, using predictions instead of original traits results in lower variability, which is directly related to the predictive performance of the model adopted. The results were almost identical for the two spectrometers, and were in agreement with the findings of Cecchinato et al. [10]. On the other hand, there was not such a close association between the decrease in additive genetic variability and the R^2^ of the external validations of the calibration models, particularly with the Vis-NIR spectrometer (Fig. 2). As a consequence, neither the heritabilities of the predictions nor their loss in additive genetic variation compared with the measured traits displayed a consistent relationship with the predictive performance of the calibration models. These findings, which are in agreement with Cecchinato et al. [10], confirm that meat quality traits predicted from infrared spectra with moderate or even low accuracy may also be heritable, and that there is potential to exploit the genetic variability.

### Correlations between laboratory-measured and abattoir-predicted meat quality traits, and their possible use for genetic improvement

The genetic correlations between the infrared-predicted and laboratory-measured traits are important in determining the effectiveness of their use as indicator traits for selective breeding [21, 27]. The degree of genetic gain achievable with indirect selection is affected by the genetic correlations between the desired and indicator traits [28]. For all the color traits, the genetic correlations between the measured traits and the Vis-NIRS predictions were extremely high, as were those between the measured traits and the Micro-NIRS predictions, despite the absence of the visible wavelengths of the spectrum. These results are consistent with the findings of the previous study [10], where the genetic correlations between the bench-top NIRS predictions of meat color traits and the corresponding measurements ranged from 0.85 to 0.99. The genetic correlations between measured and Vis-NIRS predictions for a*, b*, C* and h* were equal to 1 despite the reduced σ^2^_a_ for the predictions.

For PL, the estimated genetic correlations obtained in the present study with both instruments were substantial, and larger than in the previous study. Similarly, the Vis-NIRS predictions of pH had rather high genetic correlations with the measurements, whereas the corresponding correlations with the Micro-NIRS predictions were only moderate. In the previous study [10], CL and WBSF also proved to be difficult traits to predict from NIR spectra from the perspective of genetic improvement, as both the estimated phenotypic and genetic correlations with their laboratory measurements were inconsistent, even though the spectra were acquired from the same material, at the same location and on the same day as the laboratory analyses. These traits exhibited a partially different pattern in the present study. Although the predictive abilities of the calibration models were low, the Vis-NIRS predictions correlated well with the measured traits from the genetic standpoint, whereas the corresponding correlations with the Micro-NIRS were weaker. However, given the considerable decrease in the additive genetic variance in the Vis-NIRS predictions of WBSF, only the predictions of CL obtained from this instrument seem to be useful for selection purposes.

**Fig. 3:**
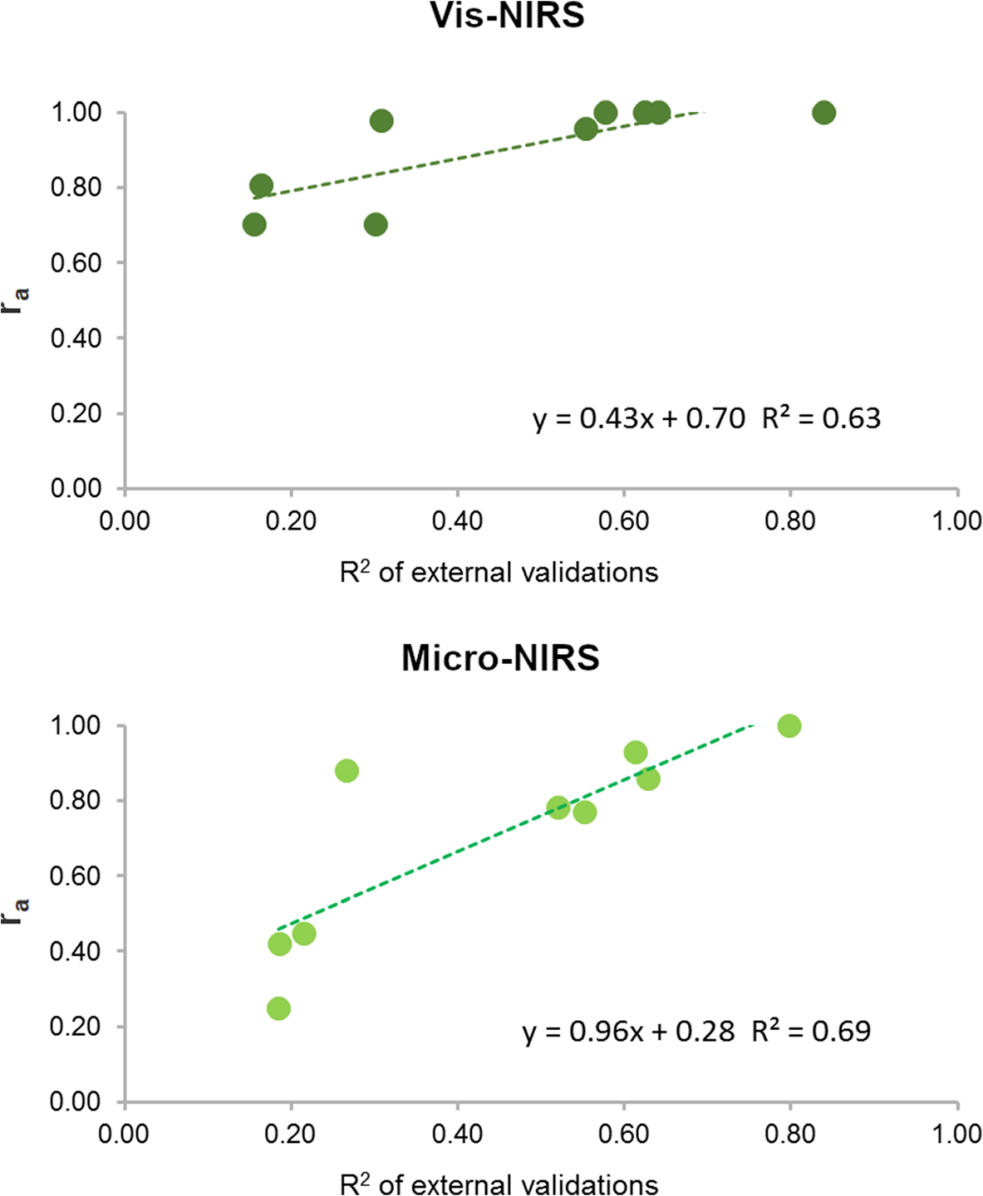
Relationship between R^2^ of external validations of predicted meat quality traits and the genetic correlation (r_a_) of predictions with the laboratory measured traits

As with the earlier findings [10], the genetic correlations between the predicted and measured traits were positively associated with the predictive ability of the calibration models (Fig. 3), but to a lesser extent than the phenotypic or residual correlations. Traits that are very accurately predicted by calibration models always show high genetic correlations with their measurements, while there is greater variability in the genetic correlations when the prediction performance is moderate or poor [22].

The use of infrared predicted traits at the population level for genetic purposes has been shown to be possible for some milk traits [29], but the use of phenotypic predictions obtained with imprecise methods is the subject of much debate. It worth noting that, in response to criticisms of a technique for predicting enteric methane emissions, Bovenhuis, van Engelen, and Visker [30] recently stated that “… even if measurements are inaccurate, imprecise, or biased, they might provide valuable information for selective breeding”, and that “When given the choice, accurate and unbiased measurements are preferred. However, such measurements are seldom available on a large scale and at reasonable cost. … However, inaccurate and biased sniffer methane phenotypes do not automatically imply inaccurate and biased methane breeding values”. When evaluating the usefulness of infrared predictions for genetically improving the quality of animal products, it should be considered that the heritability of the predictions and particularly their genetic correlations with measured traits are probably more important than phenotypic accuracy, the precision of the technique, and the lack of bias in it. A poor prediction performance of calibration model certainly affects phenotypic accuracy of NIRS predictions, but not necessarily their usefulness for the prediction of the genetic component of the same traits. In this regard, both infrared spectrometers proved to be useful tools for establishing programs for indirect genetic improvement of most of the meat quality traits, that cannot directly be measured and improved at population level.

## Conclusions

This study investigated the feasibility of selection for meat quality traits using NIRS predictions obtained from spectra acquired in the abattoir from the intact muscle surface using portable instruments. The accuracy of the predictions was good for color traits, but low for the other traits investigated. Nevertheless, the estimated genetic parameters showed that NIRS predictions of color traits, pH and PL can be used as indicator traits of the corresponding measurements for selection purposes. The results for CL were more doubtful, while the estimates for WBSF predictions were unreliable.

The two spectrometers compared in this study yielded similar results for the prediction of color traits and their relative genetic parameters. However, the Vis-NIRS instrument produced better estimates of the genetic parameters of the other predicted meat quality traits.

On the whole, the selection of complex traits such as those related to meat quality, which are difficult to phenotype directly, can benefit from the application of NIRS technology.

## Data Availability

The datasets used and/or analysed during the current study are available from the corresponding author on reasonable request.
